# Randomized phase II trial of urethral sparing intensity modulated radiation therapy in low-risk prostate cancer: implications for focal therapy

**DOI:** 10.1186/1748-717X-7-82

**Published:** 2012-06-09

**Authors:** Jeffrey Vainshtein, Eyad Abu-Isa, Karin B Olson, Michael E Ray, Howard M Sandler, Dan Normolle, Dale W Litzenberg, Kathryn Masi, Charlie Pan, Daniel A Hamstra

**Affiliations:** 1 Department of Radiation Oncology, University of Michigan Hospital, Ann Arbor, MI, USA; 2 Department of Radiation Oncology, Cedars-Sinai Medical Center, Los Angeles, CA, USA

**Keywords:** Urethral-sparing IMRT, Focal therapies, Low risk prostate cancer, Urinary quality-of-life

## Abstract

**Background:**

Low-risk prostate cancer (PCa) patients have excellent outcomes, with treatment modality often selected by perceived effects on quality of life. Acute urinary symptoms are common during external beam radiotherapy (EBRT), while chronic symptoms have been linked to urethral dose. Since most low-risk PCa occurs in the peripheral zone (PZ), we hypothesized that EBRT using urethral sparing intensity modulated radiation therapy (US-IMRT) could improve urinary health-related quality of life (HRQOL) while maintaining high rates of PCa control.

**Methods:**

Patients with National Comprehensive Cancer Network (NCCN) defined low-risk PCa with no visible lesion within 5 mm of the prostatic urethra on MRI were randomized to US-IMRT or standard (S-) IMRT. Prescription dose was 75.6 Gy in 41 fractions to the PZ + 3–5 mm for US-IMRT and to the prostate + 3 mm for S-IMRT. For US-IMRT, mean proximal and distal urethral doses were limited to 65 Gy and 74 Gy, respectively. HRQOL was assessed using the Expanded Prostate Cancer Index (EPIC) Quality of Life questionnaire. The primary endpoint was change in urinary HRQOL at 3 months.

**Results:**

From June 2004 to November 2006, 16 patients were randomized, after which a futility analysis concluded that continued accrual was unlikely to demonstrate a difference in the primary endpoint. Mean change in EPIC urinary HRQOL at 3 months was −0.5 ± 11.2 in the US-IMRT arm and +3.9 ± 15.3 in the S-IMRT arm (p = 0.52). Median PSA nadir was higher in the US-IMRT arm (1.46 vs. 0.78, p = 0.05). At 4.7 years median follow-up, three US-IMRT and no S-IMRT patients experienced PSA failure (p = 0.06; HR 8.8, 95% CI 0.9–86). Two out of 3 patients with PSA failure had biopsy-proven local failure, both located contralateral to the original site of disease.

**Conclusions:**

Compared with S-IMRT, US-IMRT failed to improve urinary HRQOL and resulted in higher PSA nadir and inferior biochemical control. The high rate of PSA failure and contralateral local failures in US-IMRT patients, despite careful selection of MRI-screened low-risk patients, serve as a cautionary tale for focal PCa treatments.

## Background

Standard treatments for localized prostate cancer include external beam radiation therapy (EBRT), brachytherapy (BT), radical prostatectomy (RP), and active surveillance. For low risk patients, oncologic outcomes between EBRT, BT, and RP appear to be similar, while the treatment-related toxicity profiles differ significantly [1-4].

Following EBRT, 10%–36% of patients report changes in urinary symptoms, which are mostly irritative or obstructive in nature [4-6]. With dose-escalated EBRT, genitourinary (GU) toxicity rates do not plateau, but become more frequent with increasing dose [5,6]. In over 1500 patients treated at Memorial Sloan-Kettering Cancer Center, Zelefsky et al. reported acute and late GU symptoms in 37% and 20% who received 81 Gy with intensity modulated radiation therapy (IMRT), compared with rates of 22% and 12% in those receiving lower doses with non-IMRT technique, respectively [5]. In the GETUG 06 randomized trial of 80 Gy vs. 70 Gy, Beckendorf et al. similarly reported grade 2 or greater late GU toxicity in 17.5% in the 80-Gy arm compared to 10% in the 70-Gy arm [6]. Despite increased urinary toxicity, dose-escalated EBRT has become standard of care based on multiple randomized trials demonstrating improvements in biochemical control [6-9].

Late urinary toxicity following RT may be due to bladder damage, sphincter damage, and urethral strictures [10-12]. Urinary strictures are seen with prostate brachytherapy, and the high-dose regions within the prostate predict the site of future strictures. Merrick et al. demonstrated that strictures were more likely to develop in those who received a higher minimum dose to the membranous urethra, with the higher dose volume distal to the prostatic apex also correlating with stricture formation [13]. These data suggest that selective treatment of the prostate and its sub-structures to different dosages may prevent RT-associated late urinary toxicity [11].

Standard IMRT (S-IMRT) for prostate cancer delivers a relatively uniform dose of radiation across the entire prostate, including the urethra. Prostate adenocarcinoma, however, tends to arise in a non-uniform anatomic distribution, with the majority (75–90%) of prostate cancers arising within the peripheral zone, while the transitional and central zones are involved in only 20–25% and 4-8% of cases, respectively [14,15]. Given the proximal periurethral location of these low risk zones, we hypothesized that selectively reducing the dose to the intraprostatic urethra in carefully selected patients would decrease urinary toxicity while maintaining high rates of disease control. We conducted a randomized Phase II study to compare the ability of urethral sparing IMRT techniques (US-IMRT) to reduce urinary side effects in patients with low-risk prostate cancer and no evidence of periurethral disease on MRI.

## Methods

### Eligibility

Patients were enrolled on an IRB approved prospective randomized Phase II trial of standard IMRT vs. US-IMRT for low-risk prostate cancer. Enrollment was limited to patients with histologically confirmed adenocarcinoma of the prostate with low-risk features by National Comprehensive Cancer Network (NCCN) criteria (Gleason score ≤ 6, PSA ≤ 10, and tumor stage T1c-T2a.) Patients were excluded if they were currently on androgen deprivation therapy, receiving any other investigations drugs, or unable to tolerate an MRI.

All patients underwent pelvic at MRI at 1.5 Tesla using a surface coil to obtain localizer, axial T1, axial T2, axial combined T1-T2, and coronal and sagittal T2-weighted sequences. Patients with evidence of a prostatic lesion within 5 mm of the proximal prostatic urethra were excluded, unless the suspected lesion was located within the peripheral zone, which would be within full-dose RT target area in both treatment arms. Eligible patients were randomized to either S-IMRT or US-IMRT using a computer randomization program. All patients underwent ultrasound-guided placement of three to five 2 mm gold fiducial prostate markers for radiation therapy daily image guidance.

### Treatment procedures

**Figure 1:**
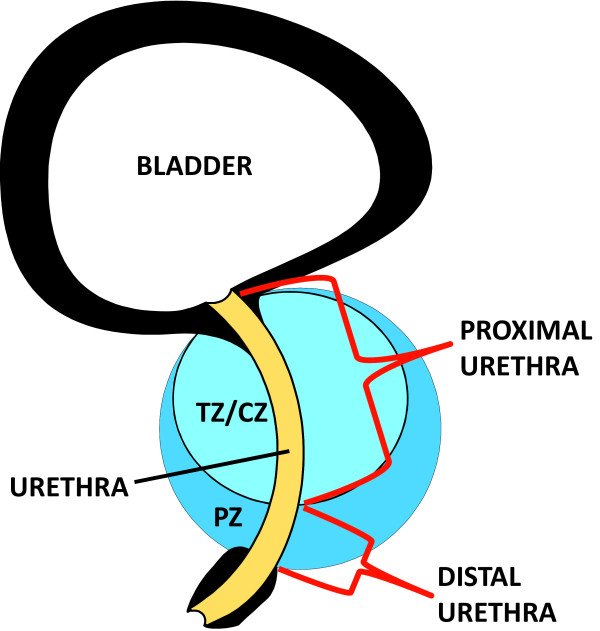
**Prostatic Sub-structure Anatomy. Mid-sagittal view of the prostatic anatomy.** Proximal urethra is defined from the bladder to the inferior-most aspect of the transitional zone/central zone (TZ/CZ) and peripheral zone (PZ) border. Distal urethra is defined from the TZ/CZ – PZ border to the prostate/pelvic floor border.

EBRT treatment utilized CT simulation in the same supine treatment position as the MRI, with the two scans aligned using mutual information registration, as previously described [16]. The target and prostatic substructure volumes, including the proximal and distal prostatic urethra, combined transitional and central zones (TZ-CZ), peripheral zone, any apparent prostate lesions, and the GU diaphragm, were defined primarily based on the MRI. The proximal prostatic urethra was defined as the portion of prostatic urethra extending superiorly from the bladder to the inferior-most aspect of the TZ-CZ/peripheral zone border, while the distal prostatic urethra was defined from the TZ-CZ/peripheral zone border superiorly to the prostate/pelvic floor border inferiorly (Figure 1). Defined organs at risk (OARs) were defined primarily with CT information, and included the bladder, rectum, GU diaphragm, femoral heads, and penile bulb. The rectum was defined from the bottom of the ischial tuberosities inferiorly to the sacral prominence superiorly. For S-IMRT, the planning target volume (PTV) the MRI-defined prostate volume expanded by 3 mm. The PTV was prescribed 75.85 Gy in 41 fractions. PTV optimization constraints included mean dose of 100% ± 3% of the prescription dose, minimum dose ≥ 93% of the prescription dose to ≥ 0.5 cc, and maximum dose (D_max_) ≤ 115% of the prescription dose (to ≥ 0.5 cc). IMRT optimization constraints for OARs were based on those used in RTOG P-0126 (http://www.rtog.org/ClinicalTrials/ProtocolTable/StudyDetails.aspx?study=0126), and included the following: for bladder, V80Gy ≤ 15%, V75Gy ≤ 25%, V70Gy ≤ 35%, and V65Gy ≤ 50%; for rectum, V75Gy ≤ 15%, V70Gy ≤ 25%, V65Gy ≤ 35%, and V60Gy ≤ 50%; for GU diaphragm, mean dose ≤ 65 Gy and maximum dose ≤115% of mean dose (to 0.5 cc); for femoral heads, mean dose ≤ 50 Gy and V52 Gy ≤ 10%; for penile bulb, mean dose ≤ 52.5 Gy and V70Gy ≤ 15%.

For US-IMRT, the PTV was the MRI-defined prostatic peripheral zone expanded uniformly by 3–5 mm, depending on clinical assessment of each case. The MRI-defined prostatic substructures were expanded uniformly by 3 mm to account for organ motion and setup uncertainties. The prescription dose and optimization goals for US-IMRT planning were the same as for S-IMRT; additional prostatic substructure OAR constraints for the proximal prostatic urethra were mean dose ≤65 Gy and D_max_ to at least 0.5 cc ≤115% of mean dose, and for the distal prostatic urethra were mean dose ≤74 Gy and D_max_ to at least 0.5 cc ≤115% of mean dose. During treatment, daily pre-treatment orthogonal imaging was used with re-positioning for variations of ≥ 3 mm. Patients were seen at least weekly during radiation therapy with documentation of treatment tolerance and prescription medications recorded.

### HRQOL assessment

Health-related quality of life (HRQOL) was assessed using the Expanded Prostate Index Composite (EPIC)[17]. EPIC questionnaires were administered at baseline, during the 3rd and 6th week of treatment, and then at 3, 6, 9, 12, 18, 24, 30, and 36 months following completion of RT.

### Followup

Patients were seen in followup with physical exam and PSA every 3 months for the first year and every 6 months thereafter for 3 years. After year 3, patients were seen in routine clinical followup, typically every 6 – 12 months.

### Statistical design

The primary endpoint was change in EPIC urinary HRQOL domain summary score at 3 months after the completion of RT (ΔEPIC-U_3mo_). At the time this protocol was designed, data from our institution demonstrated a 15–point average decline in urinary HRQOL following EBRT. A clinically significant improvement in domain symptoms was defined as ½ SD (8.75 points). In order to demonstrate a 9 point improvement with 80% statistical power using a two-sided *t*-test at a level of significance of 0.05, accrual was targeted at 63 patients on each arm, for a total of 126 patients.

Secondary endpoints included PSA failure, time to PSA failure, time to local progression, time to non-local progression, and overall survival. PSA failure after radiation was defined by the Phoenix definition of PSA nadir plus 2 ng/ml. Local progression was defined histologically as presence of prostatic carcinoma upon biopsy or an increase in palpable abnormality. Additional secondary endpoints were changes in EPIC score for bowel, sexual, and hormonal domains over time, and the rate of medical or procedural interventions between treatment groups. Time to all endpoints was calculated from the beginning of RT.

Baseline characteristics, PSA nadir, mean time to PSA nadir, and mean change in EPIC scores between treatment arms were compared using student’s *t*-test. Kaplan-Meier log-rank statistics and hazard ratios were used to compare PSA failure and time to PSA failure between US-IMRT and S-IMRT. Fisher’s exact test was used to compare the proportion of patients requiring new or increased medications for management of urinary symptoms during treatment. A p-value threshold of 0.05 was considered statistically significant. All statistical analyses were performed using Graphpad Prism version 5.01 for Windows, Graphpad Software, San Diego California USA, http://www.graphpad.com.

## Results

### Patients

From June 2004 through November 2006, 16 patients were enrolled and randomized, 8 to US-IMRT and 8 to S-IMRT. No patients were excluded due to periurethral tumor lesion location on MRI. Due to slower than expected accrual, an unplanned futility analysis was conducted in January 2007, which determined that due to the slow accrual rate, lower than expected baseline urinary EPIC scores, and smaller than expected changes in urinary EPIC score in both treatment arms, continued enrollment was unlikely to demonstrate a difference in the primary endpoint, and thus the data safety monitoring board recommended closure of the study. Baseline characteristics were well balanced between the patients in the two groups (Table 1). Baseline EPIC urinary scores were 86.9 ± 13.4 and 81.6 ± 16.5 in the US-IMRT and S-IMRT arms, respectively, which was notably lower than the anticipated baseline score of 95. Median followup was 4.8 years (range 4.0–5.8).

**Table 1:** Baseline Patient Characteristics

	**Urethral Sparing IMRT**	**Standard IMRT**	**Total Cohort**
**# of patients**	8	8	16
**Age** (mean ± SD)	63.9 ± 7.3	64.5 ± 7.1	64.21 ± 6.9
**Pretreatment PSA** (ng/mL)	8.1 ± 1.3	7.4 ± 1.5	7.74 ± 0.62
**# positive cores** (mean ± SD)	1.6 ± 0.7	1.1 ± 0.4	1.4 ± 0.6
**EPIC Urinary Domain Summary score** (mean ± SD)	86.9 ± 13.4	81.6 ± 16.5	84.3 ± 14.8
**EPIC Bowel Domain Summary score** (mean ± SD)	96.6 ± 5.1	92.5 ± 7.1	94.5 ± 6.4
**EPIC Sexual Domain Summary score** (mean ± SD)	52.5 ± 12.4	47.4 ± 32.8	50.0 ± 24.1
**EPIC Hormonal Domain Summary score** (mean ± SD)	89.3 ± 15.3	90.6 ± 9.8	90.0 ± 12.4
**Satisfaction Score** (mean ± SD)	85.7 ± 19.7	85.0 ± 13.7	85.4 ± 16.7

### Treatment

**Figure 2:**
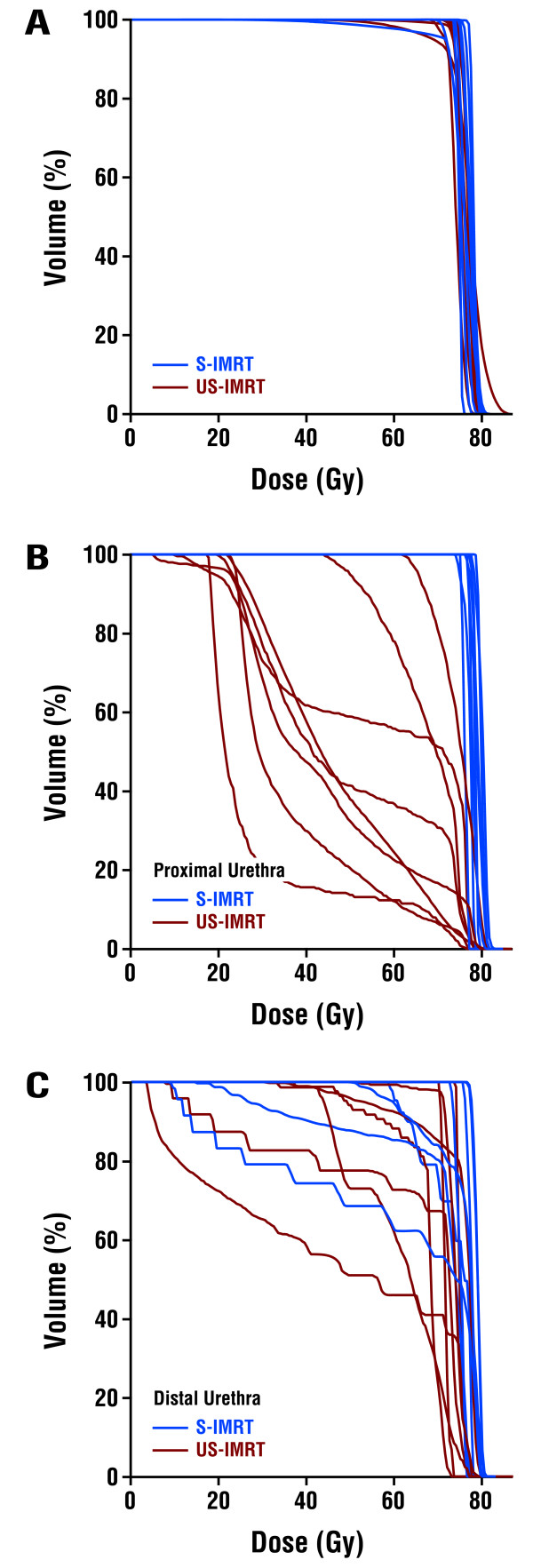
Dose-Volume Histograms for (A) Planning Target Volume, (B) Proximal Urethra, and (C) Distal Urethra for Patients Treated with S-IMRT and US-IMRT.

**Figure 3:**
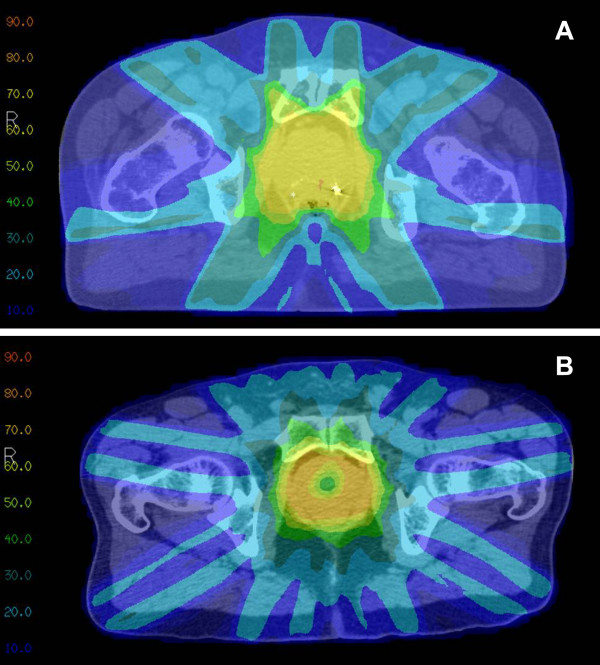
Representative dose distributions in Gray for (A) S-IMRT and (B) US-IMRT.

All 16 patients completed EBRT per randomization. The mean dose and minimum dose to 98% (D98%) of the prostate for S-IMRT were 76.3 ± 1.4 Gy and 72.4 ± 6.3 Gy, respectively. The mean dose and D98% to the peripheral zone for US-IMRT were 76.0 ± 1.0 Gy and 71.5 ± 4.5 Gy, respectively (p > 0.05 compared to S-IMRT for both mean dose and D98%). For the proximal urethra, mean doses were 76.6 ± 1.3 Gy for S-IMRT and 48.9 ± 14.7 Gy for US-IMRT (p < 0.001), with D2% of 78.4 ± 1.6 Gy and 75.8 ± 1.8 Gy, respectively (p = 0.008). For the distal urethra, mean doses were 72.9 ± 6.7 Gy for S-IMRT and 65.9 ± 9.6 Gy for US-IMRT (p = 0.12), with D2% 78.9 ± 1.6 Gy for S-IMRT and 76.2 ± 2.3 Gy for US-IMRT arm (p = 0.017). For bladder, mean dose was 26.5 Gy ± 14.5 for S-IMRT and 14.1 ± 6.9 Gy for US-IMRT (p = 0.047), while D2% was 73.9 Gy ± 2.8 for S-IMRT and 48.3 Gy ± 15.6 for US-IMRT (p < 0.001). US-IMRT reduced the mean dose to the proximal and distal urethra by 36.2% and 9.6%, respectively, compared with S-IMRT. Sparing of the proximal urethra as an OAR also decreased dose to the adjacent bladder neck, resulting in lower mean dose and D2% for bladder in the US-IMRT arm. Composite dose-volume histograms for the prostate, proximal urethra, and distal urethra are shown in Figure 2. Representative axial CT slices with dose distributions are shown in Figure 3.

### HRQOL outcomes

**Figure 4:**
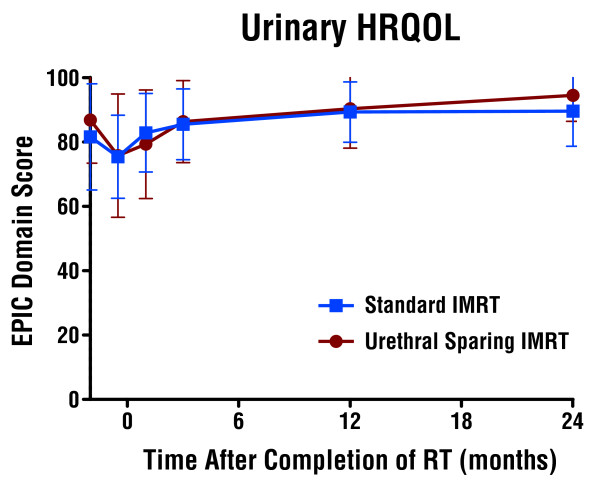
Urinary HRQOL by EPIC Urinary Domain Summary Score after Urethral Sparing IMRT and Standard IMRT.

Mean change in EPIC urinary domain HRQOL summary score at 3 months was −0.52 ± 11.2 in the US-IMRT arm and +3.98± 15.3 in the S-IMRT arm (p = 0.52). No differences were seen in either the mean change in EPIC urinary domain HRQOL summary or subset scores during RT or at the 1, 3, 12 or 24 month timepoints (Figure 4 & Table 2). There were also no differences between arms in the bowel, sexual, hormonal, or satisfaction domain scores at 3, 12, and 24 months (Table 2).

**Table 2:** EPIC HRQOL Domain Summary Scores for Patients Treated with US-IMRT and S-IMRT

	**US-IMRT** (mean ± S.D.)	**S-IMRT** (mean ± S.D.)	**Total Cohort** (mean ± S.D.)
EPIC URINARY DOMAIN			
EPIC-Urine*_6wkRT_	75.8 ± 19.1	75.5 ± 12.9	75.6 ± 15.6
Change^†^EPIC-U_6wkRT_	-11.1 ± 12.8	-6.2 ± 11.9	-8.6 ± 12.2
**EPIC-Urine**_**1mo**_	79.4 ± 16.9	82.9 ± 12.2	81.1 ± 14.3
Change EPIC-U_1mo_	-7.6 ± 13.8	1.3 ± 12.2	-3.1 ± 13.4
**EPIC-Urine**_**3mo**_	86.4 ± 12.7	85.5 ± 11.0	85.9 ± 11.5
Change ΔEPIC-U_3mo_	-0.5 ± 11.2	3.9 ± 15.3	1.7 ± 13.2
**EPIC-Urine**_**1yr**_	90.4 ± 12.2	89.3 ± 9.4	89.9 ± 10.5
Change EPIC-U_1yr_	3.5 ± 4.7	7.7 ± 10.2	5.6 ±8.0
**EPIC-Urine**_**2yr**_	94.5 ± 8.1	89.6 ± 10.9	92.1 ± 9.6
Change EPIC-U_2yr_	7.6 ± 8.8	8.0 ± 10.4	7.8 ± 9.3
EPIC BOWEL DOMAIN			
**EPIC-Bowel**^**††**^_**3mo**_	95.1 ± 4.5	88.5 ± 14.1	91.8 ± 10.8
Change EPIC-B_3mo_	-1.6 ± 7.2	-3.9 ± 9.6	-2.7 ± 8.3
**EPIC-Bowel**_**1yr**_	97.4 ± 5.1	89.8 ± 16.27	93.6 ± 12.3
Change EPIC-B_1yr_	0.8 ± 5.3	-2.6 ± 11.56	-0.9 ± 8.9
**EPIC-Bowel**_**2yr**_	96.6 ± 4.45	89.6 ± 12.7	93.1 ± 9.9
Change EPIC-B_2yr_	0.0 ± 3.0	-2.9 ± 10.4	-1.4 ± 7.5
EPIC SEXUAL DOMAIN			
**EPIC-Sex**^**§**^_**3mo**_	49.2 ± 17.1	46.8 ± 29.2	48.0 ± 23.2
Change EPIC-S_3mo_	-3.3 ± 10.8	-0.6 ± 12.8	-2.0 ± 11.5
**EPIC-Sex**_**1yr**_	43.0 ± 20.1	47.4 ± 25.5	45.2 ± 22.3
Change EPIC-S_1yr_	-9.6 ± 15.5	0.0 ± 13.4	-4.8 ± 14.8
**EPIC-Sex**_**2yr**_	39.5 ± 21.4	47.9 ± 32.7	43.7 ± 27.0
Change EPIC-S_2yr_	-13.0 ± 21.6	0.5 ± 5.97	-6.3 ± 16.8
EPIC HORMONAL/VITALITY DOMAIN			
**EPIC-Hormonal**^**||**^_**3mo**_	90.4 ± 14.2	92.2 ± 8.9	91.3 ± 11.5
Change EPIC-H_3mo_	1.0 ± 4.6	1.6 ± 5.4	1.3 ± 4.9
**EPIC-Hormonal**_**1yr**_	89.3 ± 14.8	93.0 ± 7.6	91.1 ± 11.5
Change EPIC-H_1yr_	0.0 ± 6.8	2.3 ± 4.8	1.2 ± 5.8
**EPIC-Hormonal**_**2yr**_	95.3 ± 5.5	95.6 ± 4.9	95.4 ± 5.1
Change EPIC-H_1yr_	6.0 ± 10.3	5.0 ± 5.6	5.5 ± 8.0
EPIC SATISFACTION DOMAIN			
**EPIC-Satisfaction**^**¶**^_**3mo**_	96.4 ± 9.5	90.0 ± 13.7	84.4 ± 25.6
Change EPIC-Sat_3mo_	10.7 ± 19.7	5.0 ± 20.9	8.3 ± 19.5
**EPIC-Satisfaction**_**1yr**_	92.9 ± 12.2	95.0 ± 11.2	93.8 ± 8.3
Change EPIC-Sat_1yr_	7.1 ± 12.2	10.0 ±13.7	8.3 ± 12.3
**EPIC-Satisfaction**_**2yr**_	100 ± 0	95.0 ± 11.2	97.9 ± 12.5
Change EPIC-Sat_2yr_	14.3 ± 19.7	10.0 ± 13.7	12.5 ± 16.9

*U = urinary; ^†^Δ = change from baseline; ^††^B = bowel; ^§^S = sexual; ^||^H = hormonal; ^¶^Sat = satisfaction

A positive number for change reflects an improvement in HRQOL while a negative number for change reflects a decline in HRQOL. No comparisons between S-IMRT and US-IMRT were statistically different to a p-value <0.05.

Thirty-eight percent of US-IMRT arm patients and 63% of S-IMRT arm patients either initiated or increased the dose of alpha-blockers during treatment (p = 0.62). One patient in the US-IMRT arm developed urinary retention requiring self-catheterization at 3.6 Gy, which resolved prior to completion of RT. Of note, urinary EPIC score improved from 72.3 at baseline to 91.7 at 3 months in this patient.

Four patients in each arm had a decline in urinary domain HRQOL summary score during RT, which returned to baseline in 75% by 3 months and in 100% by 1 year. At 2 years, 2 patients in the US-IMRT arm and 3 patients in the S-IMRT arm reported improved urinary HRQOL domain summary scores compared with baseline, whereas the remaining patients all reported stable urinary HRQOL compared to their baseline pre-treatment scores.

### Treatment efficacy

**Figure 5:**
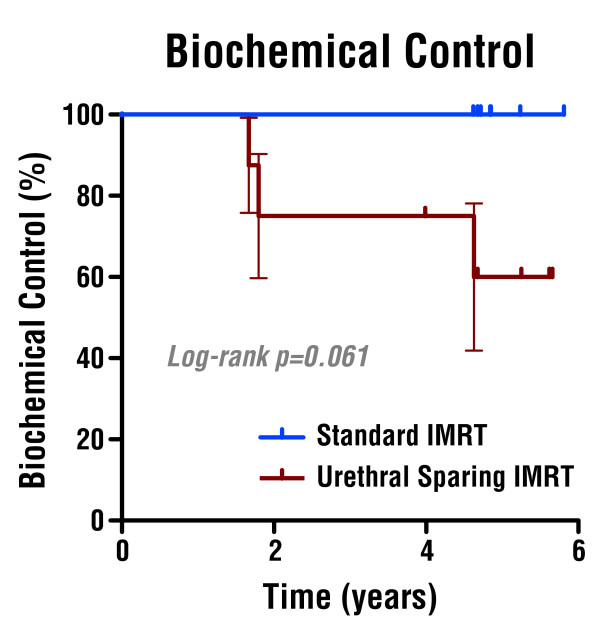
Biochemical Control after Urethral Sparing IMRT and Standard IMRT.

Mean PSA nadir was higher in the US-IMRT arm than in the S-IMRT arm (1.5 vs. 0.78 ng/ml, p = 0.05). There were no differences in time to PSA nadir between US-IMRT and S-IMRT (2.5 vs. 2.8 years, p > 0.1). Three patients treated with US-IMRT and 0 patients treated with S-IMRT experienced PSA failure, with a two-year PSA failure rate of 25% in the US-IMRT arm and 0% in the S-IMRT arm (p = 0.06, log-rank; HR 8.8, 95% CI 0.9–86; Figure 5). The 3 patients who experienced PSA failure underwent biopsy at 2.2, 3.1, and 4.4 years following completion of RT, 2 of which returned positive (one with and one without treatment effect). Both biopsy proven recurrences were located in the peripheral zone contralateral to the original site of disease and were not within the region of the intentionally spared prostate gland. No distant failures or deaths occurred in either arm.

## Discussion

Our randomized study failed to demonstrate an improvement in urinary HRQOL in low-risk prostate cancer patients treated with US-IMRT compared with S-IMRT. Furthermore, and unexpectedly, a higher rate of PSA failure was observed in the US-IMRT arm (37.5% vs. 0%, p = 0.06). It is striking that despite the very small number of randomized patients, a quite large difference in biochemical control was nonetheless able to be detected. That this difference was of only borderline statistical significance should not diminish the impact of our findings, given the low power of such a small study to detect a statistical difference.

There are a number of potential explanations for the higher rate of BF observed with US-IMRT. One such explanation is that limiting the PTV to the MRI-defined PZ allowed progression of radiographically occult adenocarcinoma or prostatic intraepithelial neoplasia in the TZ-CZ. Similarly, underdosing of the CZ-TZ could have produced suboptimal suppression of PSA production by benign prostatic parenchyma in these non-target prostatic subsites, resulting in a physiologic PSA rise meeting criteria for BF and leading to biopsy-detection of locally persistent disease that may have otherwise remained clinically indolent. The higher PSA nadir observed in the US-IMRT arm supports both of these hypotheses. Additionally, although MRI screening was used to exclude patients with periurethral lesions, underdosing of MRI-occult periurethral disease could increase the probability of tumor control by US-IMRT, as has been recently suggested by other investigators [18]. The fact that both biopsy-proven local recurrences were located in the contralateral peripheral zone from the initial index lesion, rather than in the lower dose peri-urethral regions of the prostate, reduces the likelihood that underdosing contributed to clinical progression. The clinical significance of the contralateral local failures after US-IMRT in patients with low-risk and low-volume disease is debatable, given that such patients typically have excellent clinical outcomes, even without radical therapy, under current active surveillance protocols [19]. Nevertheless, the contralateral failures in our study, despite MRI screening and enrollment of only low risk patients, underscore the multifocal nature of prostate cancer and highlight the limitations of MRI to reliably identify patients with unifocal disease who may be appropriate candidates for focal prostate cancer therapies, a topic of ongoing debate in the prostate cancer community [20-22].

In addition to producing a higher rate of BF, US-IMRT failed to have a beneficial impact on urinary toxicity in our study. Several factors may explain this disappointing result. The baseline urinary EPIC function in our cohort was 84.3, significantly lower than the anticipated baseline score of 95. In a large comparator study, mean urinary irritative and incontinence domain-specific EPIC symptom scores were 88.2 and 92.9, respectively, compared with 83.3 and 85.9 in our study, suggesting that baseline urinary function in our small cohort of patients may not have been representative of a larger population [4]. Furthermore, the observed mean change in urinary HRQOL in the S-IMRT arm of +3.9 was lower than expected, and was actually better than in the US-IMRT arm. It is possible that the higher rate of alpha-blocker usage in S-IMRT patients may have contributed to recovery of urinary HRQOL and masked any reduction in toxicity in the US-IMRT arm.

The effect of prostate motion on urethral sparing may also have limited our ability to actually spare the uretha during treatment, despite static treatment plans suggesting adherence to stringent urethral dose constraints. For instance, one study of 427 prostate cancer patients (representing >11,000 fractions) evaluated using real-time continuous tracking of prostate motion observedintra-treatment motion in excess of 2 mm in 66% of fractions and in excess of 3 mm in 28% [23]. Similar rates have been observed in other studies [24,25]. At the time this study was initiated, daily image guidance with implanted gold fiducial markers was standard practice at our institution, and real-time intra-fractional tracking of prostate motion was not employed. It is possible, therefore, that intra-fractional prostate motion could have resulted in delivery of higher than intended radiation doses to the intraprostatic urethra and lower than intended doses to the periurethral PTV, contributing to both the lack of urinary toxicity reduction and increased rate of BF observed with US-IMRT. However, as noted above, it is unlikely that underdosing of the periurethral PTV was responsible for the observed clinical failures in our study, given that both local recurrences were located away from the periurethral region. Nonetheless, modern technology that decreases rotational setup error and intra-fractional motion uncertainty through 4D-localization and continuous real-time prostate motion tracking may reduce the risk of both of these treatment delivery errors, and could potentially improve the disappointing clinical outcomes observed with US-IMRT in our randomized study [24,25].

One notable feature of our study was that a combination of daily image-guided IMRT with small PTV margins (3–5 mm) provided excellent HRQOL across all EPIC domains. The change in EPIC urinary irritative/obstructive, urinary incontinence, bowel, sexual, and hormonal domain scores at 2 years for the entire cohort were +8.6 ± 11.8, +7.6 ± 8.5,−1.4 ± 7.5,−6.3 ± 16.8, and +5.5 ± 8.0, respectively (where a positive number represents an improvement in HRQOL), which compare favorably with previous reports by Wei et al. and Sanda et al. of HRQOL outcomes using the EPIC instrument following EBRT for prostate cancer [3,4] . Importantly, HRQOL in our cohort at 2 years was either stable or non-significantly improved compared to baseline in all domains except for the sexual domain, which was non-significantly decreased.

## Conclusions

In summary, US-IMRT failed to improve urinary HRQOL, and resulted in a higher PSA nadir and inferior biochemical control in patients treated with gold fiducial-based daily image guidance. The high rate of BF and contralateral local failures, despite careful selection of low-risk patients with MRI screening, should serve as a cautionary tale for focal prostate cancer therapies.

## Abbreviations

PCa, Prostate cancer; HRQOL, Health-related quality of life; PZ, Peripheral zone; IMRT, Intensity modulated radiation therapy; US – IMRT, Urethral-sparing IMRT; S-IMRT, Standard IMRT; EPIC, Expanded Prostate Cancer Index; QOL, Quality of life; EBRT, External beam radiation therapy; BT, Brachytherapy; RP, Radical prostatectomy; GU, Genitourinary; TZ-CZ, Combined transitional and central zones; OARs, Organs at risk; PTV, Planning target volume; Dmax, Maximum dose; HRQOL, Health related quality of life.

## Competing interests

There are no actual or potential conflicts of interest to disclose for any of the authors.

## Authors’ contributions

JV reviewed and analyzed the data, performed statistical analyses, created the figures, and drafted the manuscript. EAI reviewed and analyzed the data, performed statistical analyses, and assisted in drafting the manuscript. KO was responsible for long term clinical followup of patients enrolled on the clinical trial and data collection. MR designed and enrolled patients to the clinical trial. HS designed and enrolled patients to the clinical trial. DN performed the statistical design and analysis. DL performed dosimetric analysis for the manuscript. KM performed dosimetric analysis for the manuscript. CP was the principal investigator of the clinical trial. DH was responsible for long term follow up of patients enrolled on the clinical trial and assisted in the design and drafting of the manuscript with final approval of manuscript. All authors read and approved the final manuscript.
